# Validation of an automated chromogenic *in situ* hybridization protocol for detection of cytomegalovirus in formalin-fixed, paraffin-embedded renal graft biopsies

**DOI:** 10.1590/2175-8239-JBN-2024-0255en

**Published:** 2026-04-17

**Authors:** Juliana Vitor Rangel, Janice Mery Chicarino de Oliveira Coelho, Lilimar da Silveira Rioja, Luís Cristóvão Porto, Andréa Monte-Alto-Costa

**Affiliations:** 1 Universidade do Estado do Rio de Janeiro, Faculdade de Medicina, Departamento de Patologia e Medicina Laboratorial, Rio de Janeiro, RJ, Brazil.; 2 Universidade do Estado do Rio de Janeiro, Núcleo Tecnológico em Reparo Tecidual e Histocompatibilidade, Rio de Janeiro, RJ, Brazil.

**Keywords:** Kidney, Biopsy, Transplant, Cytomegalovirus, In situ hybridization.

## Abstract

**Introduction:**

Histopathological diagnosis of human cytomegalovirus (HCMV) infection in formalin-fixed, paraffin-embedded (FFPE) tissues stained with hematoxylin–eosin relies on the identification of characteristic cytopathic changes, including eosinophilic intranuclear and cytoplasmic viral inclusions. Chromogenic in situ hybridization (CISH) enables the localization of specific nucleic acid sequences in histological sections, increasing diagnostic sensitivity. Automated CISH platforms allow standardized and reproducible detection of viral RNA or DNA in FFPE tissues. This study aimed to validate an automated CISH protocol for the detection of HCMV in multiple tissue types, including renal allograft biopsies processed at the Anatomical Pathology Division of UERJ.

**Methods:**

Two groups of FFPE samples were analyzed. The first group included ten samples from various tissues, including kidney, palate, esophagus, stomach, and colon; nine positive and one negative for HCMV by immunohistochemistry (IHC). The second group included twenty renal allograft biopsies; nineteen without previous diagnosis and one positive by IHC. For CISH, fluorescein-conjugated oligonucleotide probes targeting HCMV RNA expressed during the early replication phase were used, with hybridization and detection performed on an automated platform.

**Results:**

In Group 1, all samples, including the case previously negative by IHC, were positive for HCMV by CISH. In Group 2, six of the twenty renal biopsies were positive, including the sample already identified as positive by IHC.

**Conclusions:**

The automated CISH protocol demonstrated high sensitivity and reproducibility for HCMV detection, supporting its validation and use in the diagnosis of renal biopsies and other tissues, as well as its incorporation into the routine workflow of Anatomical Pathology laboratories.

## Introduction

Human cytomegalovirus (HCMV) is a DNA virus (β-herpesvirus) that replicates within the nucleus of infected cells^1^. Infection typically occurs through direct contact with infected bodily fluids, aerosols from coughing, sneezing, or speaking, saliva, blood transfusions, sexual contact, or vertical transmission. Although HCMV is prevalent worldwide, most infected individuals remain asymptomatic^2^. Following infection, the virus can establish lifelong latency and reactivate in transplant recipients and immunosuppressed individuals due to weakened immune defenses^3^. Reactivation can result in severe infections in transplant patients and individuals with HIV, making HCMV a significant cause of morbidity and mortality in these groups^1,2^.

In transplant patients undergoing immuno­suppression, HCMV infection can exacerbate graft rejection. Diagnosis is typically performed using serological tests, pp65 antigenemia assays, and nucleic acid amplification methods, such as the polymerase chain reaction (PCR)^4,5,6^. In cases of suspected “compartmentalized” disease, where the virus is undetectable in blood samples, a biopsy may be indicated^7^.

HCMV can also be identified in histopathological analyses of formalin-fixed, paraffin-embedded tissues using hematoxylin and eosin (HE) staining, immunohistochemistry (IHC), and *in situ* hybridization (ISH)^8,9,10^. In HE-stained slides, the characteristic histopathological finding of HCMV infection is the presence of “owl’s eye” inclusion bodies in infected cells and cytomegaly. However, these features are typically visible only when there is a high viral load^11,12^.

In post-transplant renal biopsies, the presence of eosinophilic intranuclear and cytoplasmic viral inclusions may be accompanied by an inflammatory infiltrate of mononuclear cells and neutrophils, as well as necrosis of infected cells^13^. In such cases, histological techniques are highly specific methods for determining HCMV involvement in the affected organ.

Additionally, tissue samples suspected of HCMV infection do not always exhibit the characteristic morphological features described in the literature. Instead, they may display atypical cytopathic effects, such as karyomegaly without intranuclear inclusions or eosinophilic cytoplasmic granules^14,15^. In these cases, diagnosis is confirmed using alternative techniques, such as immunohistochemistry (IHC) and *in situ* hybridization (ISH), which are highly sensitive and specific methods, allowing for detection even in the presence of low viral loads^7,16^.

Chromogenic *in situ* hybridization (CISH) is a technique that enables the localization of specific nucleic acid sequences within histological sections. Until the late 1990s, both IHC and ISH techniques, despite their increased sensitivity for detecting HCMV, were performed manually. This manual approach resulted in extended processing times, potential errors or technical variability, and required human intervention at every stage of the procedure. To address these limitations, automation of IHC and ISH techniques was developed^4,8^. Automation ensures consistent labeling quality, improved standardization, operational optimization, traceability, lower costs, and enhanced biosafety^9,17,18^. CISH has proven suitable for routine laboratory use due to the simplicity of its execution and the ease of interpreting results^19^.

The objective of this study was to validate an automated CISH protocol for the detection of HCMV in various types of tissue fixed in 10% formalin and embedded in paraffin, including renal grafts, from the Pathology Department of the Rio de Janeiro State University.

## Methods

For this study, two groups of samples were used. The first group consisted of 10 samples from different tissue types (Table 1); nine were diagnosed as positive for HCMV by immunohistochemistry (IHC), and one had a clinical history suggestive of HCMV infection but without confirmation by HE staining or IHC. The second group comprised 20 samples from renal graft biopsies collected between 2022 and 2023, including 19 samples with no prior histopathological diagnosis of HCMV and one sample confirmed as positive by IHC (Table 2). The study was approved by the Research Ethics Committee of the Rio de Janeiro State University (UERJ) (protocol no. 6.811.172).

**Table 1:** Samples for cish evaluation

Case	Sex	Organ	HE positivity	Histopathological	IHC positivity
1	F	palate	yes	ENI	yes
2	M	kidney	yes	MII	yes
3	M	kidney	no	MII	no
4	M	rectum	yes	ENI+MII	yes
5	M	esophagus	yes	MII	yes
6	F	stomach	yes	FNII	yes
7	M	kidney	yes	MII	yes
8	M	esophagus	yes	MII	yes
9	M	colon	yes	ENI+MII	yes
10	F	stomach	yes	FNII	yes
11	M	kidney	no	ABS	no

Abbreviations – ENI: eosinophilic nuclear inclusion; MII: mononuclear inflammatory infiltrate; FNII: fibrino-neutrophilic inflammatory infiltrate; ABS: absence of HCMV characteristics.

**Table 2:** R enal biopsies selected for cish technique testing

Case	Sex	Clinical indication
12	M	Rejec + Cell
13	F	Rejec + Cell
14	F	Rejec + Cell
15	M	Clin
16	M	Rejec + Cell
17	F	Clin + Rejec + Cell
18	M	Clin + Rejec + Cell
19	F	Clin + Rejec + Cell
20	M	Rejec + Cell
21	F	Clin + Rejec + Cell
22	M	Rejec + Cell
23	F	Clin + Rejec + Cell
24	F	Rejec + Cell
25	M	Rejec + Cell
26	M	Rejec + Cell
27	F	Rejec + Cell
28	M	Rejec + Cell
29	M	Rejec + Cell
30	F	Rejec + Cell
31	F	Clin + Rejec + Cell

Abbreviations – Cell: cellular alterations under microscopy; Clin: clinical suspicion of infection; Rejec: nonspecific rejection.

The samples included in the first group exhibited clinical suspicion and/or morphological alterations consistent with human cytomegalovirus (HCMV) infection, as observed in HE-stained histological sections, such as intranuclear inclusion bodies. The second group consisted of retrospective renal graft biopsies collected between 2022 and 2023, which had not undergone specific diagnostic testing for HCMV due to the unavailability of appropriate techniques at the time the biopsies were performed. However, these samples exhibited clinical suspicion of viral infection or histopathological features suggestive of HCMV infection, such as cellular enlargement (cytomegaly).

For HCMV diagnosis using IHC, an anti-CMV monoclonal mouse antibody (clone DDG9/CCH2; Cell Marque, Rocklin, CA, USA) was employed on the BOND-MAX system (Leica Biosystems, Bannockburn, Scotland). Positive cells in IHC-stained slides were quantified according to the scoring system described below.

The samples were fixed in 10% buffered formalin during the pre-analytical phase and processed using a tissue processor (LUPETEC PT2, São Paulo, Brazil) with paraffin maintained at 60 ºC. Following histological processing, the samples were embedded in paraffin blocks.

For the CISH procedure, histological sections were prepared using a manual microtome (LEICA RM2125 RTS; Leica Biosystems), cut at 3 µm, and mounted on positively charged, silanized histological slides (Bond Plus Slides, Leica Biosystems). The slides included a positive control tissue section (palate – sample 1, Table 1) and a negative control tissue section (kidney – sample 11, Table 1). The sections were prepared on the same day as each reaction to ensure the integrity of the RNA present. The reactions were performed in triplicate on alternate days.

A fluorescein-conjugated oligonucleotide probe, the BOND Ready-to-Use ISH HCMV Probe PB0614 (Leica Biosystems; Newcastle, UK), was used. This probe is designed for the qualitative identification of RNA copies of the early gene (IE72) of HCMV in formalin-fixed, paraffin-embedded tissue samples. The reaction was revealed using a chromogenic CISH method (described below). For process automation, the BOND-MAX Leica Biosystems device was used, following the manufacturer’s instructions.

To assess RNA preservation and reaction specificity, each round included two slides of positive control tissue (palate) with two different probes: the PB0785 RNA Positive Control Probe (Leica Biosystems, Newcastle, UK), which detects RNA preservation in cells, and the PB0809 RNA Negative Control Probe (Leica Biosystems, Newcastle, UK), a single oligonucleotide derived from zebra DNA used to confirm that the tissue sequence has no homology with any human sequence. Both probes were fluorescein-labeled and followed the same detection procedure as the other oligonucleotide probes used in the study on the BOND-MAX equipment.

The CISH procedure for the selected samples included the following steps: manual slide labeling, preparation of histological sections, deparaffinization, and drying of slides in an oven at 60 °C for 1 hour. Slides were then labeled with the equipment’s designated identifiers and covered with Bond Universal Covertiles (Leica Biosystems). This preparation process took approximately 2 hours. Subsequently, the slides were loaded into the device according to the manufacturer’s instructions.

For the first group of samples, the procedures performed using the automated system were carried out according to the manufacturer’s instructions for the BOND-MAX instrument (Leica Biosystems), as detailed below (Protocol 1). The reagents used in the reactions were supplied by Leica Biosystems.

1. Deparaffinization with Bond Dewax Solution at 72 °C for 5 minutes;
2. Washing in absolute alcohol for 1 minute, followed by 4 minutes in distilled water;
3. Tissue pre-treatment (RNA target unmasking) with Bond Enzyme 1 at 37 °C for 15 minutes;
4. Three washes in Bond Wash Solution for 2, 2, and 1 minute each;
5. RNA hybridization for 2 hours at 37 °C using BOND Ready-to-Use ISH HCMV Probe PB0614;
6. Three washes in Bond Wash Solution for 2, 2, and 1 minute each;
7. Incubation with peroxidase inhibitor for 6 minutes at room temperature;
8. Three washes in Bond Wash Solution for 2, 2, and 1 minute each;
9. Incubation with rat anti-fluorescein monoclonal antibody AR0833 (Leica Biosystems) for 15 minutes at room temperature;
10. Three washes in Bond Wash Solution for 2, 2, and 1 minute each;
11. Incubation with post-primary reagent for 8 minutes at room temperature, followed by a 6-minute wash in Bond Wash Solution;
12. Incubation with linkage polymer for 8 minutes at room temperature, followed by a 4-minute wash in Bond Wash Solution and 1 minute in distilled water;
13. Reaction revelation with DAB (3,3’-diaminobenzidine tetrahydrochloride) for 7 minutes at room temperature, followed by a 2-minute distilled water wash;
14. Counterstaining with hematoxylin for 5 minutes at room temperature, followed by a 1-minute wash in Bond Wash Solution and a 30-second wash in distilled water.

For renal graft biopsies (Table 2), another protocol was employed to reduce damage due to enzyme treatment and background signal. All steps were performed using the automated BOND-MAX system (Leica Biosystems) according to the manufacturer’s instructions. The following modifications were implemented (Protocol 2):

- After rinsing with distilled water, tissue pretreatment (RNA target retrieval) was conducted using BOND Epitope Retrieval Solution pH 9 for 30 minutes at 99 °C;
- Slides were incubated with a peroxidase inhibitor for 30 minutes;
- Slides were incubated with a protein-blocking inhibitor for 30 minutes at room temperature;
- Incubation with rat monoclonal anti-fluorescein antibody AR0833 (Leica Biosystems) was performed for 11 minutes at room temperature;
- Development with DAB (3,3’-diaminobenzidine tetrahydrochloride) was performed for 1 minute at room temperature.

The automated CISH procedure took approximately 4.5 hours. After the process was completed, the slides were removed from the device, hydrated in graded ethanol (4 times), and cleared in xylene (3 times) before mounting in acrylic resin (Allklan, Allkimia) with a coverslip.

The RNA-positive and RNA-negative control tissue slides followed the same protocol as the test samples, with differences in step 3, using specific fluorescein-conjugated oligonucleotide probes for positive and negative mRNA transcripts.

The slides were analyzed separately under an optical microscope by two pathologists (JMCOC and LSR) and one biologist (JVR). Positivity was defined as intense brown, cytoplasmic, granular, or nuclear immunoreactivity. Positive cells were quantified by examining the entire slide. The reproducibility of positive staining was evaluated by comparative analysis of the three slides from each block in the first group (Table 1). Samples not exhibiting the specified characteristics were considered negative.

Slides were semiquantitatively evaluated using the following scoring system: (0) no positive cells; (1) 1 to 5 positive cells; (2) 6 to 11 positive cells; and (3) more than 12 positive cells. The entire sample was examined to determine the final score.

## Results

**Figure 1:**
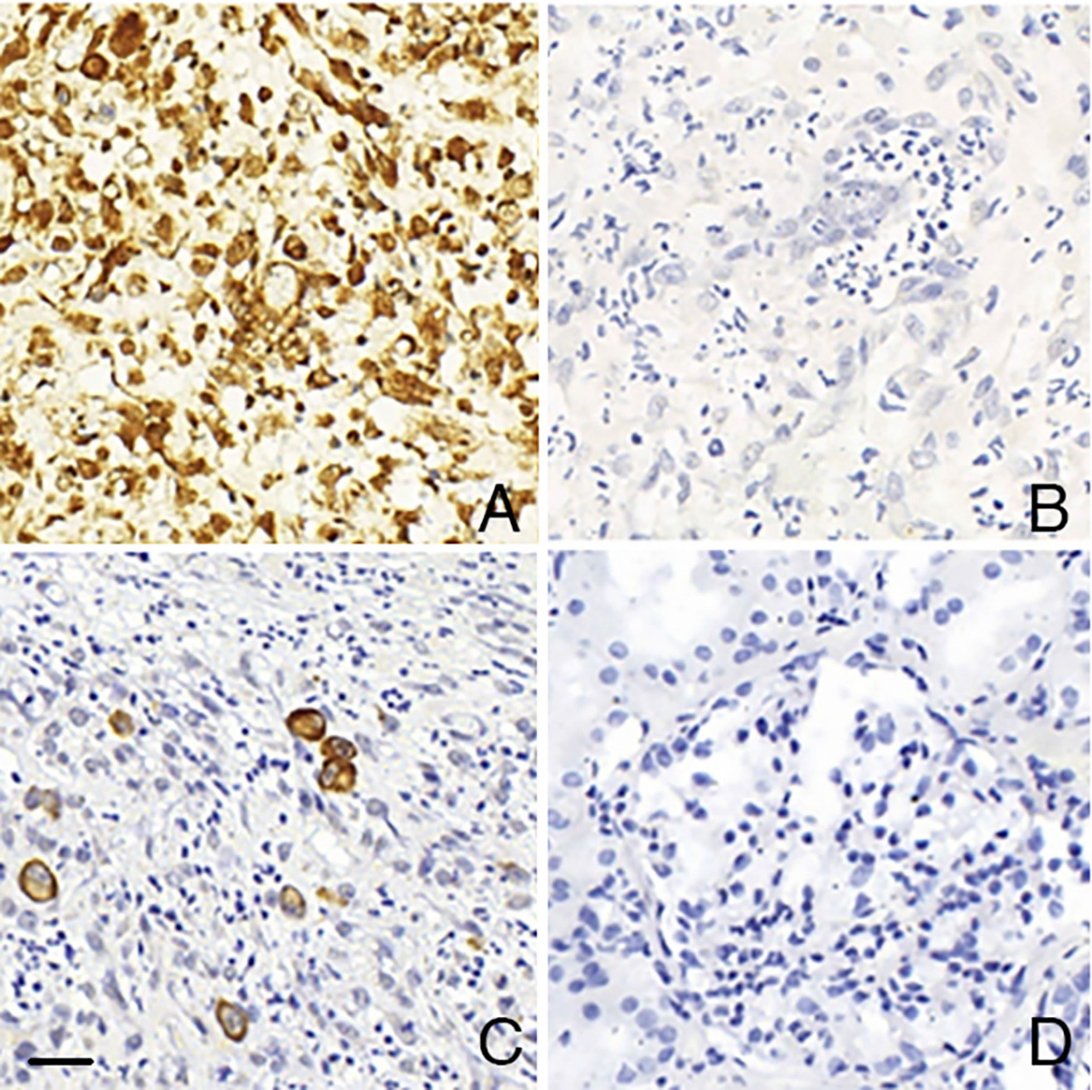
Verification of RNA integrity and standard controls for the CISH technique for HCMV. A – Palatal tissue showing RNA expression throughout the tissue using the PB0785 RNA Positive Control Probe. B – Palatal tissue showing no RNA expression throughout the tissue using the PB0809 RNA Negative Control Probe. C – Palatal tissue with cells exhibiting cytoplasmic and nuclear granular cytomegalic RNA expression (Positive Control – Standard). D – Renal tissue without cytomegalic RNA expression in cells (Negative Control – Standard). Scale bar: 20 µm.

**Figure 2:**
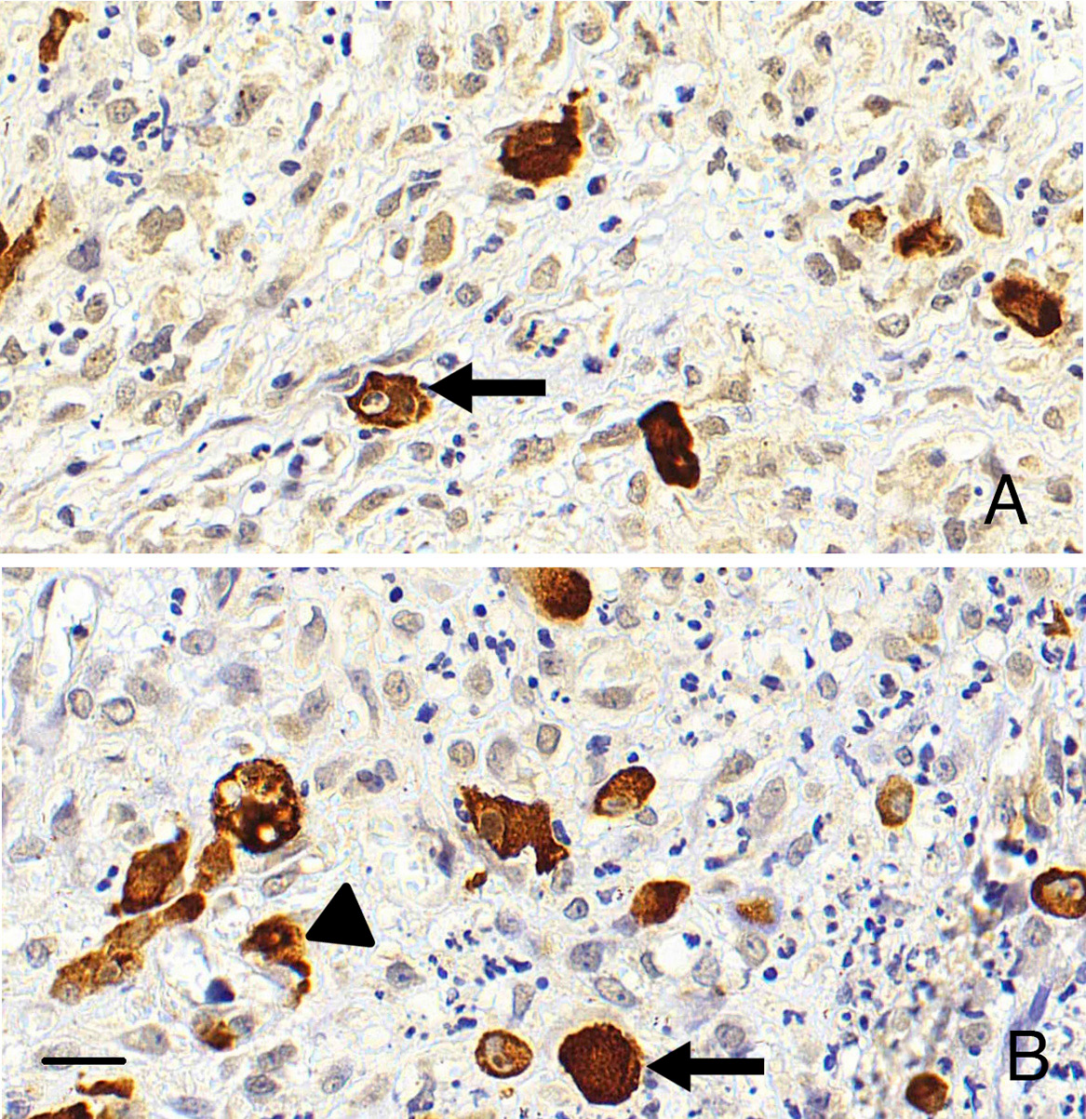
CISH staining patterns of HCMV-positive cells in palatal tissue. A – Cytoplasmic positivity (arrow). B – Nuclear positivity (arrow) and granular positivity (arrowhead). Scale bar: 20 µm.

RNA preservation was confirmed by the positive reactions observed when using the PB0785 RNA Positive Control Probe (Figure 1A). Reaction specificity was verified by the absence of positive results when the PB0809 RNA Negative Control Probe was used (Figure 1B). All slides with positive control tissue showed positivity (Figure 1C), while none of the negative control slides showed positivity (Figure 1D). HCMV-positive reactions were detected with granular, cytoplasmic, and nuclear labeling in infected cells (Figure 2).

**Figure 3:**
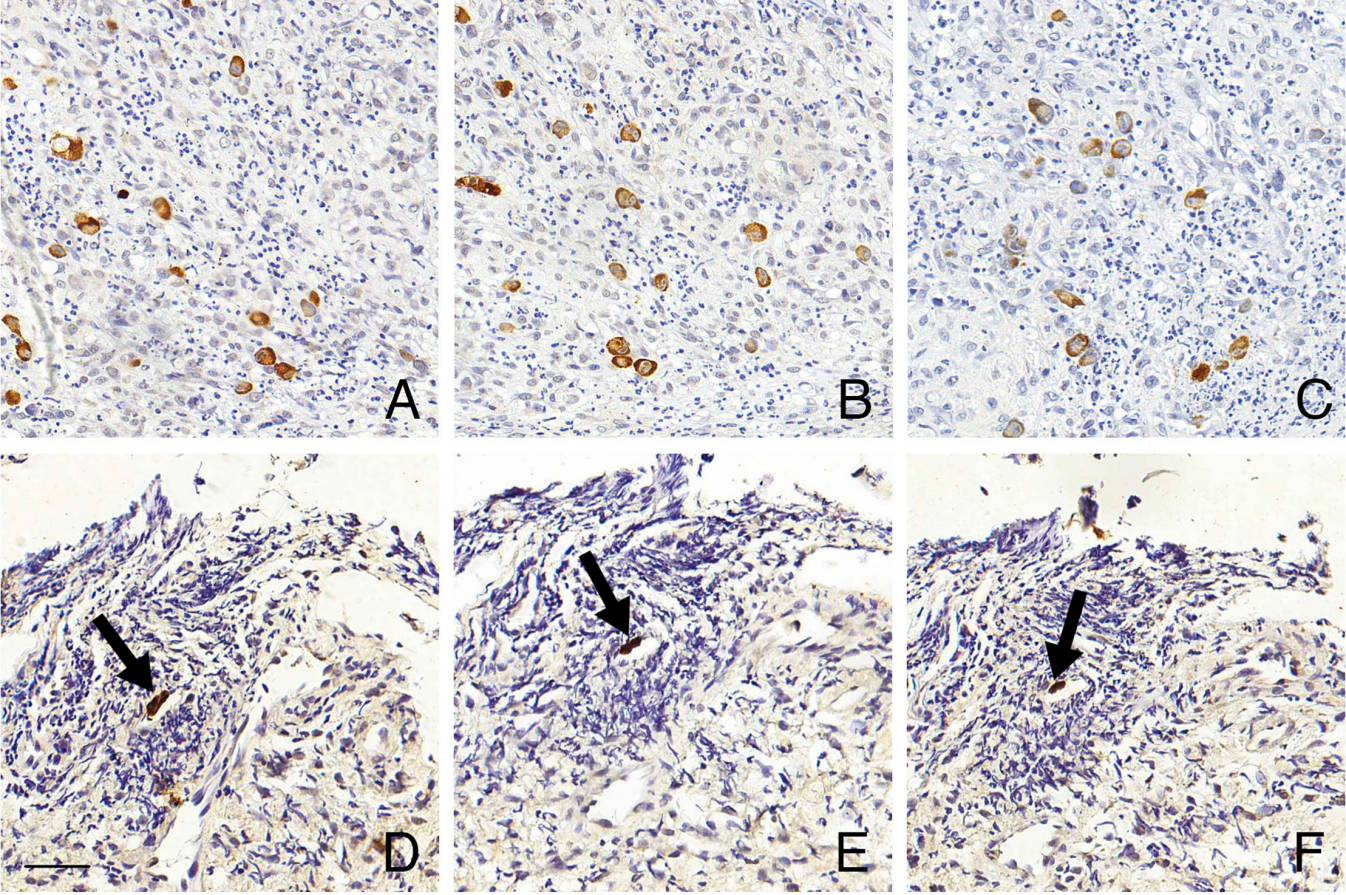
Reproducibility of automated CISH for HCMV detection. Triplicate chromogenic *in situ* hybridization (CISH) reactions demonstrating consistent detection of cytomegalovirus (HCMV) RNA expression. A, B, and C – Same region of a palatal biopsy; D, E, and F – Same region of an esophageal biopsy, positive cell (arrow). Scale bar: 50 µm.

The results of the first group of 10 samples are presented in Table 3. The CISH reaction in this group was reproducible, with the same region exhibiting positivity across all three slides tested on different days (Figure 3).

**Table 3:** C omparison of the quantitative analysis of hcmv using ihc and cish techniques

Case	IHC – Score	CISH – Score		
		test 1	test 2	test 3
1	3	3	3	3
2	1	1	1	1
3	0	2	2	2
4	1	1	1	1
5	1	1	1	1
6	3	3	3	3
7	1	1	1	1
8	1	1	1	1
9	1	1	1	1
10	1	1	1	1
11	0	0	0	0

Note – Score: (0) no positive cells; (1) 1 to 5 positive cells; (2) 6 to 11 positive cells; and (3) more than 12 positive cells.

**Figure 4:**
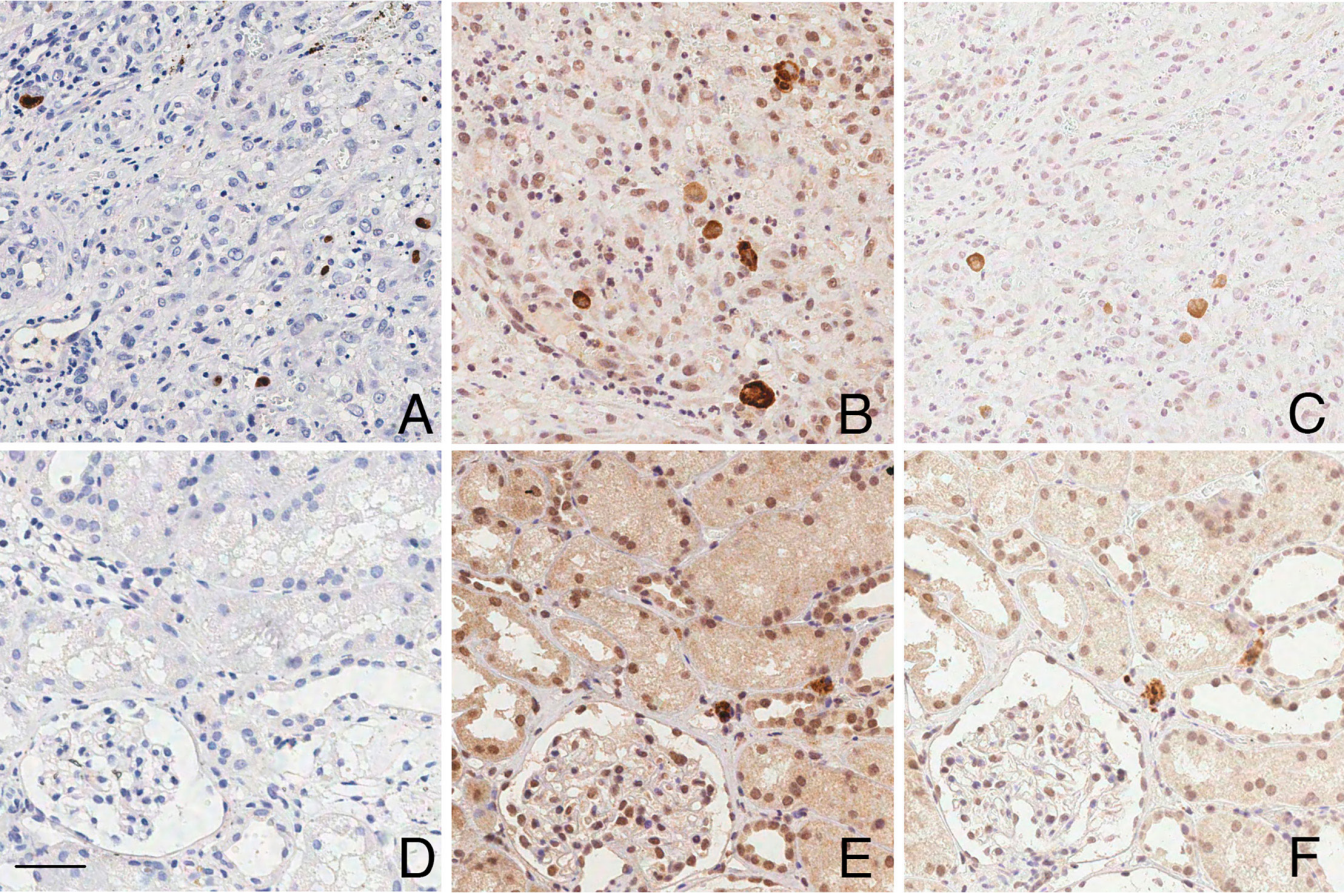
Comparative analysis of CISH protocols for HCMV detection in palatal and renal biopsies. A and D – IHC technique positive for HCMV protein expression in cells in palatal (A) and negative for kidney biopsy (D); B and E – CISH technique (Protocol 1) positive for HCMV RNA expression in palatal (B) and in renal biopsy - interstitium of the renal tubular epithelium (E); C and F – CISH technique (Protocol 2) positive for HCMV RNA expression in palatal (C) and in renal biopsy - interstitium of the renal tubular epithelium (F). Note that with protocol 2 the CISH background is reduced. Scale bar: 20 µm.

**Figure 5:**
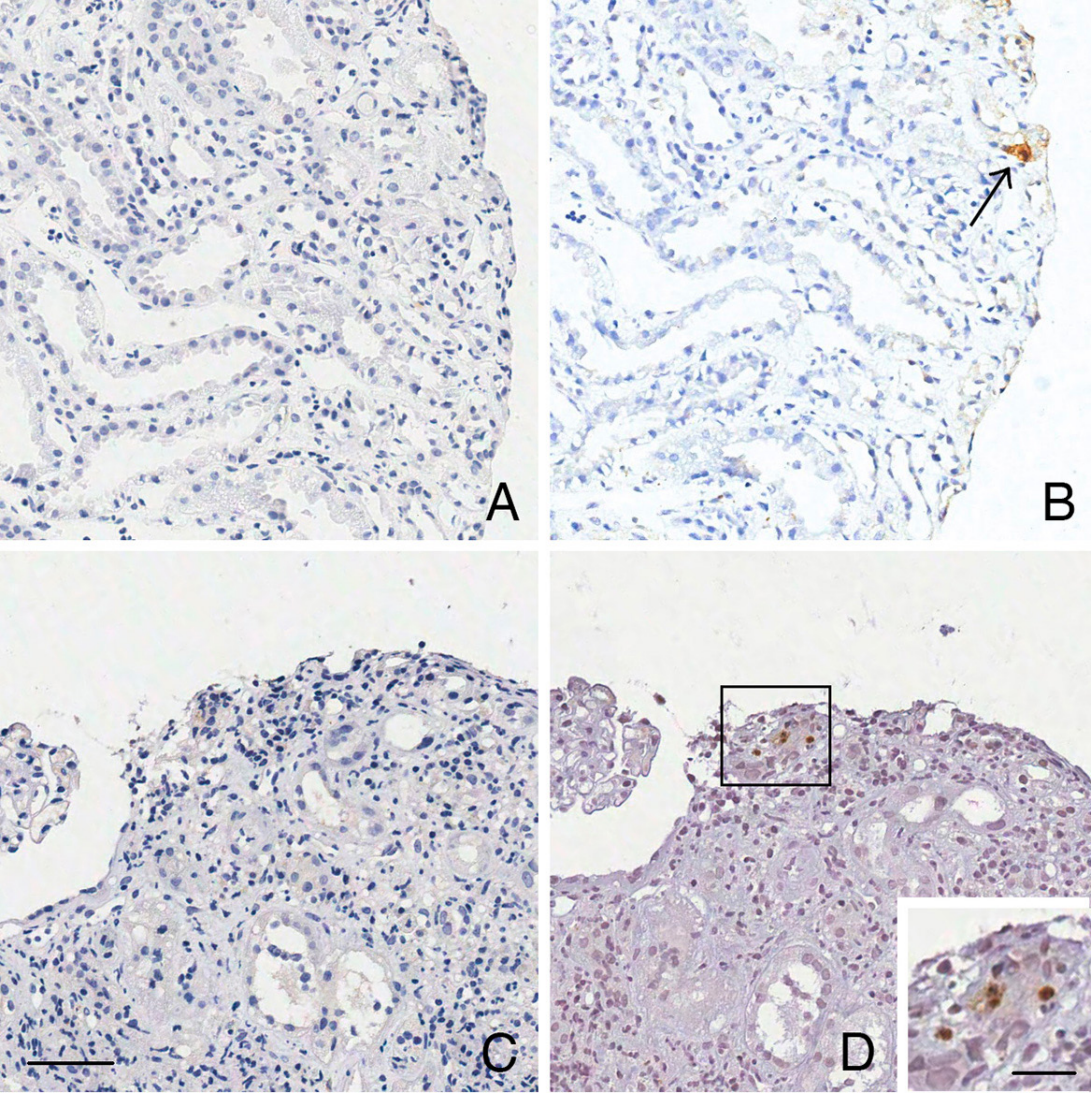
Comparison of IHC and CISH techniques for HCMV detection in renal graft biopsies. A and C – IHC technique negative for HCMV protein expression in renal cells. B and D – Similar regions showing CISH-positive cells for HCMV RNA. Scale bar: 50 µm; inset 20 µm.

The results from the renal biopsies are presented in Table 4. The IHC reaction was positive in only one sample. The CISH reaction was positive in seven samples and negative in 13 out of the 20 selected samples. A comparison of immunohistochemistry (IHC) and CISH showed that cells negative in IHC exhibited positivity in CISH (Figures 4 and 5). The modifications in the CISH protocol (Protocol 2) reduced the background and improved the overall quality of labeling (Figures 4 B-C and E-F).

**Table 4:** Comparison of the quantitative analysis of hcmv using ihc and cish techniques in renal biopsies

Case	IHC – score	CISH – score
12	0	0
13	0	0
14	0	0
15	0	0
16	0	0
17	0	0
18	0	2
19	0	3
20	0	3
21	0	0
22	0	0
23	0	0
24	0	3
25	0	0
26	0	2
27	0	3
28	0	0
29	0	0
30	0	0
31	1	3

Note – Score: (0) no positive cells; (1) 1 to 5 positive cells; (2) 6 to 11 positive cells; and (3) more than 12 positive cells.

Some cells that were negative for IHC labeling of HCMV were positive when CISH was employed (Figure 5).

## Discussion

This study aimed to validate and implement an automated *in situ* hybridization (CISH) protocol for HCMV detection, using samples of various tissue types, including renal tissue from transplant patients. The technique selected was CISH with RNA detection during the early phase of viral replication. This choice was based on the availability of the equipment required to perform this specific CISH protocol, on the fact that it was the only commercially available probe for HCMV detection in paraffin-embedded tissues in the Brazilian market at the time of the study, and on its potential as a complementary method to the automated IHC technique, which is performed on the same equipment. Based on the results obtained, it will be possible to implement automated HCMV detection as part of the routine diagnostic workflow. Although IHC is frequently the standard technique for detecting HCMV in biopsies, CISH offers several advantages, as demonstrated in Table 5.

**Table 5:** Comparison between ihc and cish for hcmv detection in biopsies

	IHC	CISH
Sensitivity	++	++++
Cost	$$	$$$
Time (manual)	16h	16h
Time (automated)	5h	5h
Indication	Suspected infection	Suspected infectionIHC negative or score 1

CISH, similar to *in situ* hybridization (ISH), allows the assessment of genetic abnormalities such as chromosomal number changes, translocations, or amplifications through the hybridization of DNA (or RNA) probes labeled with complementary sequences in target tissue interphase nuclei and is applicable to formalin-fixed, paraffin-embedded tissues^19^. CISH is also comparable to fluorescence *in situ* hybridization (FISH) regarding pretreatments and hybridization protocols but differs in the detection method; CISH uses a chromogenic reaction based on peroxidase, like IHC, instead of fluorescent markers. RNA or DNA probes conjugated to fluorescein are detected indirectly using an enzyme-conjugated antibody (peroxidase). The enzymatic reaction with the chromogenic substrate (DAB) results in permanent brown signals that are visible with standard optical microscopy, allowing long-term slide storage. In contrast, FISH requires time-sensitive interpretation, a fluorescence microscope for reading, and has shorter slide durability, making CISH a cost-effective alternative^20^. The comparative analysis between the two methods, FISH and CISH, was clearly presented and correlated by Hsi et al.^21^. While both techniques have distinct advantages, CISH has increasingly proven to be a practical, cost-effective, and reliable alternative to FISH. CISH is suitable for the diagnosis and detection of RNA molecules in various tissue samples, including HCMV, often linked to neoplastic tissues and inflammatory lesions^10^. CISH allows the simultaneous evaluation of gene amplification and tissue morphology, with slides that can be stored for extended periods and visualized with optical microscopy^19^.

Automated CISH is widely used in Pathology Departments^22-24^ to address the limitations of the manual method, such as long execution time, overnight incubation, and the need for continuous technician involvement^17,22,23,25^. Automation in IHC, ISH, and CISH ensures consistent quality, standardized operations, cost efficiency, ease of use, and enhanced biosafety^18^.

The HCMV oligonucleotide probe conjugated to fluorescein was used to detect HCMV through RNA expressed during early viral replication. Nakajima et al.^26^ demonstrated that RNA oligonucleotide probes are the most sensitive and suitable for detecting low transcript levels, which explains the variation in cell labeling observed in our results, as shown in Table 2. Some tested samples showed different cell counts in CISH compared to prior IHC, which was consistent across all three reactions. Other samples did not show significant differences in cell counts between the two techniques, possibly due to low viral copy numbers^7,16^.

The second protocol, using BOND Epitope Retrieval Solution pH 9 for tissue pretreatment (RNA target unmasking), was validated (Figure 5) as an alternative for potentially enzyme-sensitive samples due to pre-analytical issues such as tissue degradation observed in HE staining or challenges related to fixation and histological processing^27,28^.

Given the limited size of renal graft biopsy samples and the need to preserve tissue morphology, an alternative protocol (Protocol 2) was evaluated. Modifications included extended incubation time for endogenous peroxidase blocking, the addition of a protein blocking step, and a shortened DAB incubation period. These adjustments were necessary because renal tissue contains high levels of endogenous biotin, which can cause background staining and false-positive results following DAB application^29,30^.

Cytoplasmic non-specific labeling (considered negative) in some samples, alongside positively labeled cells, may indicate disrupted, poorly fixed capsid proteins, with possible hybridization of residual viral RNA, as noted by Wolber and Lloyd^31^. However, these findings did not impact the overall reaction outcome.

Automated CISH showed reproducibility, high sensitivity with positive reactions in all IHC-positive samples, and no labeling in negative controls, validating the technique for implementation. Studies by Todorovic´-Rakovic´^19^, Atabati et al.^32^, and Khaleghian et al.^33^ also confirm that the CISH method is superior when compared to FISH and IHQ, demonstrating greater efficacy, specificity, and sensitivity.

It is important to note that HCMV infection is a major cause of morbidity and mortality among immunocompromised and/or immunosuppressed patients, including solid organ transplant recipients, especially within the first six months post-transplant, due to immunosuppression. HCMV is the most common complication in transplantation, with a fivefold increased risk of overall mortality and an elevenfold increased risk of death related to HCMV infection. Thus, HCMV infection prevention and treatment are critical for transplant success^34,35^ and are associated with an increased graft rejection risk^36,37^. However, to confirm the clinical relevance and the diagnosis of CMV viral nephritis, it will be crucial to conduct an expanded study with a larger population of kidney transplant recipients, including a detailed description of kidney histology and patient outcomes, together with CISH detection^38^.

Accurate histopathological diagnosis using more sensitive techniques, such as CISH, may indicate post-transplant organ rejection due to HCMV infection or its association. Standardizing this method enables its integration into routine laboratory practices and enhances diagnostic reliability, particularly in cases of recurrent renal biopsies, aligning with findings of Rimsza et al.^8^ and international standards^39^.

## Conclusion

In our tests, CISH outperformed IHC in detecting HCMV-positive cells, especially in samples with few infected cells or atypical morphological patterns. CISH showed greater sensitivity for detecting infected cells at an early stage, expressing cytoplasmic viral RNA, regardless of full viral presentation in the cell, which is characterized by cytomegaly and intranuclear inclusions. Although variables such as fixation, histological processing, and tissue type may influence *in situ* hybridization outcomes, the protocol can be optimized and tailored to overcome these potential technical limitations. The validation and standardization of the automated CISH technique, as well as its implementation in diagnostic and research settings for HCMV in renal biopsies and other tissue types, will benefit post-transplant renal patients and other patients with clinical and/or morphological suspicion of HCMV, increasing histopathological diagnostic accuracy. It is important to highlight that this study was intended for the validation of the protocol and the standardization of the automated CISH technique. Larger studies involving the follow-up of transplant patients, the potential early detection of HCMV infection, and patient prognosis are still needed.

## Data Availability

The datasets generated and/or analyzed during the current study are available from the corresponding author upon reasonable request.
