# Supplementary material for: Validation of an automated chromogenic in situ hybridization protocol for detection of cytomegalovirus in formalin-fixed, paraffin-embedded renal graft biopsies
Source: J Bras Nefrol. 2026 Apr 17;48(3):e20240255. doi: 10.1590/2175-8239-JBN-2024-0255en (PMC13123571; doi:10.1590/2175-8239-JBN-2024-0255en)
Supplement: Translation

# Translation: Validação de um protocolo automatizado de hibridização *in situ* cromogênica para detecção de citomegalovírus em biópsias de enxerto renal fixadas em formol e incluídas em parafina

**DOI:** 10.1590/2175-8239-JBN-2024-0255pt

**Authors:** Juliana Vitor Rangel, Janice Mery Chicarino de Oliveira Coelho, Lilimar da Silveira Rioja, Luís Cristóvão Porto, Andréa Monte-Alto-Costa

**Affiliations:** 1 Universidade do Estado do Rio de Janeiro, Faculdade de Medicina, Departamento de Patologia e Medicina Laboratorial, Rio de Janeiro, RJ, Brasil.; 2 Universidade do Estado do Rio de Janeiro, Núcleo Tecnológico em Reparo Tecidual e Histocompatibilidade, Rio de Janeiro, RJ, Brasil.

**Keywords:** Rim, Biópsia, Transplante, Citomegalovírus, Hibridização in situ

## Abstract

**Introdução:**

O diagnóstico histopatológico da infecção pelo citomegalovírus humano (HCMV) em tecidos fixados em formol e incluídos em parafina (FFIP), corados por hematoxilina-eosina, baseia-se na identificação de efeitos citopáticos característicos, como inclusões virais eosinofílicas intranucleares e citoplasmáticas. A hibridização *in situ* cromogênica (CISH) permite localizar sequências específicas de ácidos nucleicos em cortes histológicos, aumentando a sensibilidade diagnóstica. Plataformas automatizadas de CISH possibilitam detecção padronizada e reprodutível de RNA ou DNA em tecidos FFIP. Este estudo teve como objetivo validar um protocolo automatizado de CISH para detecção de HCMV em diferentes tipos de tecido, incluindo biópsias de enxerto renal da Disciplina de Anatomia Patológica da UERJ.

**Métodos:**

Dois grupos de amostras FFIP foram analisados. O primeiro incluiu 10 amostras de tecidos diversos (rim, palato, esôfago, estômago e cólon), sendo nove positivas e uma negativa para HCMV por imuno-histoquímica (IHQ). O segundo incluiu 20 biópsias de enxerto renal, 19 sem diagnóstico prévio e uma positiva por IHQ. Para a CISH, foi utilizada sonda de oligonucleotídeos conjugada à fluoresceína, que detecta RNA do HCMV expresso na fase inicial da replicação viral, em equipamento automatizado.

**Resultados:**

No grupo 1, todas as amostras, incluindo a previamente negativa por IHQ, foram positivas pela CISH. No grupo 2, seis das 20 biópsias renais foram positivas, incluindo a já identificada por IHQ.

**Conclusões:**

O protocolo automatizado de CISH demonstrou alta sensibilidade e reprodutibilidade para detecção de HCMV, permitindo sua validação e aplicação no diagnóstico de biópsias renais e outros tecidos e implementação na rotina dos laboratórios de Anatomia Patológica.

## Introdução

O citomegalovírus humano (HCMV) é um vírus de DNA (β-herpesvírus) que se replica no núcleo das células infectadas^1^. A infecção ocorre geralmente por contato direto com fluidos corporais infectados, incluindo aerossóis provenientes de tosse, espirros ou fala, saliva, transfusões sanguíneas, contato sexual ou transmissão vertical. Embora o HCMV seja amplamente disseminado no mundo, a maioria dos indivíduos infectados permanece assintomática^2^. Após a infecção, o vírus pode estabelecer latência ao longo da vida e se reativar em receptores de transplantes e indivíduos imunossuprimidos, em decorrência do enfraquecimento das defesas imunológicas^3^. A reativação pode resultar em infecções graves em pacientes transplantados e indivíduos com HIV, tornando o HCMV uma importante causa de morbidade e mortalidade nesses grupos^1,2^.

Em pacientes transplantados sob imunossupressão, a infecção pelo HCMV pode agravar a rejeição do enxerto. O diagnóstico é geralmente realizado por testes sorológicos, ensaios de antigenemia pp65 e métodos de amplificação de ácidos nucleicos, como a reação em cadeia da polimerase (PCR)^4,5,6^. Em casos de suspeita de doença “compartimentalizada”, na qual o vírus não é detectado em amostras de sangue, pode ser indicada a realização de biópsia^7^.

O HCMV também pode ser identificado em análises histopatológicas de tecidos fixados em formol e incluídos em parafina, utilizando coloração por hematoxilina-eosina (HE), imuno-histoquímica (IHQ) e hibridização *in situ* (ISH)^8,9,10^. Em lâminas coradas por HE, o achado histopatológico característico da infecção por HCMV é a presença de corpúsculos de inclusão em “olhos de coruja” nas células infectadas, além de citomegalia. Entretanto, essas características geralmente são visíveis apenas quando há alta carga viral^11,12^.

Em biópsias renais pós-transplante, a presença de inclusões virais eosinofílicas intranucleares e citoplasmáticas pode ser acompanhada por infiltrado inflamatório composto por células mononucleares e neutrófilos, além de necrose das células infectadas^13^. Nesses casos, as técnicas histológicas constituem métodos de elevado grau de especificidade para determinar o envolvimento do HCMV no órgão afetado.

Além disso, amostras de tecido com suspeita de infecção por HCMV nem sempre apresentam as características morfológicas clássicas descritas na literatura. Podem, ao contrário, exibir efeitos citopáticos atípicos, como cariomegalia sem inclusões intranucleares ou grânulos citoplasmáticos eosinofílicos^14,15^. Nessas situações, o diagnóstico é confirmado por técnicas alternativas, como IHQ e ISH, as quais são métodos altamente sensíveis e específicos, permitindo a detecção mesmo na presença de baixa carga viral^7,16^.

A hibridização *in situ* cromogênica (CISH) é uma técnica que permite a localização de sequências específicas de ácidos nucleicos em cortes histológicos. Até o final da década de 1990, tanto a IHQ quanto a ISH, apesar da elevada sensibilidade para a detecção do HCMV, eram realizadas manualmente. Essa abordagem manual resultava em tempos de processamento prolongados, possíveis erros ou variabilidade técnica e exigia intervenção humana em todas as etapas do procedimento. Para superar essas limitações, foram desenvolvidos métodos automatizados para IHQ e ISH^4,8^. A automação garante qualidade consistente de marcação, melhor padronização, otimização operacional, rastreabilidade, redução de custos e maior biossegurança^9,17,18^. A CISH tem se mostrado adequada para uso rotineiro em laboratórios devido à simplicidade de execução e à facilidade de interpretação dos resultados^19^.

O objetivo deste estudo foi validar um protocolo automatizado de CISH para a detecção de HCMV em diversos tipos de tecidos fixados em formol a 10% e incluídos em parafina, incluindo enxertos renais, provenientes do Departamento de Patologia da Universidade do Estado do Rio de Janeiro.

## Métodos

Para este estudo, foram utilizados dois grupos de amostras. O primeiro grupo consistiu em 10 amostras de diferentes tipos de tecido (Tabela 1), das quais nove haviam sido diagnosticadas como positivas para HCMV por imuno-histoquímica (IHQ), e uma apresentava história clínica sugestiva de infecção por HCMV, porém sem confirmação por coloração por HE ou IHQ. Já o segundo grupo foi composto por 20 amostras de biópsias de enxerto renal coletadas entre 2022 e 2023, incluindo 19 amostras sem diagnóstico histopatológico prévio de HCMV e uma amostra confirmada como positiva por IHQ (Tabela 2). O estudo foi aprovado pelo Comitê de Ética em Pesquisa da Universidade do Estado do Rio de Janeiro (UERJ) (Protocolo nº 6.811.172).

**Tabela 1:** Amostras para avaliação por cish

Caso	Sexo	Órgão	Positividade no HE	Histopatológico	Positividade na IHQ
1	F	palato	sim	INE	sim
2	M	rim	sim	IIM	sim
3	M	rim	não	IIM	não
4	M	reto	sim	INE + IIM	sim
5	M	esôfago	sim	IIM	sim
6	F	estômago	sim	IIFN	sim
7	M	rim	sim	IIM	sim
8	M	esôfago	sim	IIM	sim
9	M	cólon	sim	INE + IIM	sim
10	F	estômago	sim	IIFN	sim
11	M	rim	não	AC	não

Abreviaturas – INE: Inclusão nuclear eosinofílica; IIM: Infiltrado inflamatório mononuclear; IIFN: Infiltrado inflamatório fibrino-neutrofílico; AC: Ausência de características de HCMV.

**Tabela 2:** Biópsias renais selecionadas para testes da técnica cish

Caso	Sexo	Indicação clínica
12	M	Rej + Cel
13	F	Rej + Cel
14	F	Rej + Cel
15	M	Clin
16	M	Rej + Cel
17	F	Clin + Rej + Cel
18	M	Clin + Rej + Cel
19	F	Clin + Rej + Cel
20	M	Rej + Cel
21	F	Clin + Rej + Cel
22	M	Rej + Cel
23	F	Clin + Rej + Cel
24	F	Rej + Cel
25	M	Rej + Cel
26	M	Rej + Cel
27	F	Rej + Cel
28	M	Rej + Cel
29	M	Rej + Cel
30	F	Rej + Cel
31	F	Clin + Rej + Cel

Abreviaturas – Cel: alterações celulares na microscopia; Clin: suspeita clínica de infecção; Rej: rejeição não específica.

As amostras incluídas no primeiro grupo apresentavam suspeita clínica e/ou alterações morfológicas compatíveis com infecção por HCMV, observadas em cortes histológicos corados por HE, tais como corpos de inclusão intranucleares. O segundo grupo consistiu em biópsias retrospectivas de enxertos renais coletadas entre 2022 e 2023, que não haviam passado por testes diagnósticos específicos para HCMV devido à indisponibilidade de técnicas apropriadas à época da realização das biópsias. No entanto, essas amostras apresentavam suspeita clínica de infecção viral ou características histopatológicas sugestivas de infecção por HCMV, como aumento celular (citomegalia).

Para o diagnóstico de HCMV por IHC, utilizou-se um anticorpo monoclonal de camundongo anti-CMV (clone DDG9/CCH2; *Cell Marque*, Rocklin, CA, EUA) processado no sistema BOND-MAX (*Leica Biosystems*, Bannockburn, Escócia). As células positivas nas lâminas coradas por IHQ foram quantificadas de acordo com a pontuação descrita a seguir.

As amostras foram fixadas em formalina tamponada a 10% durante a fase pré-analítica e processadas em processador automático de tecidos (LUPETEC PT2, São Paulo, Brasil), com a parafina mantida a 60 °C. Após o processamento histológico, as amostras foram incluídas em blocos de parafina.

Para o procedimento de CISH, cortes histológicos foram preparados utilizando micrótomo manual (LEICA RM2125 RTS; *Leica Biosystems*), seccionados a 3 µm e montados em lâminas histológicas positivas e silanizadas (*Bond Plus Slides, Leica Biosystems*). As lâminas incluíram um corte de tecido controle positivo (palato – amostra 1, Tabela 1) e um corte de tecido controle negativo (rim – amostra 11, Tabela 1). Os cortes foram realizados no mesmo dia de cada reação para garantir a integridade do RNA presente. As reações foram realizadas em triplicata, em dias alternados.

Foi utilizada uma sonda de oligonucleotídeo conjugada à fluoresceína, a sonda *BOND Ready-to-Use ISH HCMV Probe* PB0614 (*Leica Biosystems*; Newcastle, Reino Unido). Essa sonda é projetada para a identificação qualitativa de cópias de RNA do gene precoce (IE72) do HCMV em amostras de tecido fixadas em formalina e incluídas em parafina. A reação foi revelada por método cromogênico CISH (descrito abaixo). Para a automação do processo, utilizou-se o equipamento BOND-MAX da *Leica Biosystems*, seguindo as instruções do fabricante.

Para avaliar a preservação do RNA e a especificidade da reação, cada rodada incluiu duas lâminas de tecido controle positivo (palato) com duas sondas diferentes: a PB0785 *RNA Positive Control Probe* (*Leica Biosystems*, Newcastle, Reino Unido), que detecta a preservação do RNA nas células, e a PB0809 *RNA Negative Control Probe* (*Leica Biosystems*, Newcastle, Reino Unido), um oligonucleotídeo único derivado de DNA de zebra, utilizado para confirmar que a sequência tecidual não possui homologia com sequências humanas. Ambas as sondas foram marcadas com fluoresceína e seguiram o mesmo procedimento de detecção das demais sondas utilizadas no estudo no equipamento BOND-MAX.

O procedimento de CISH para as amostras selecionadas incluiu as seguintes etapas: identificação manual das lâminas, preparação dos cortes histológicos, desparafinização e secagem das lâminas em estufa a 60 °C por uma hora. Em seguida, as lâminas foram identificadas com os códigos definidos pelo equipamento e cobertas com *Bond Universal Covertiles (Leica Biosystems*). Esse processo de preparação levou aproximadamente duas horas. Posteriormente, as lâminas foram inseridas no equipamento conforme as instruções do fabricante.

Para o primeiro grupo de amostras, os procedimentos realizados no sistema automatizado seguiram as recomendações do fabricante para o equipamento BOND-MAX (*Leica Biosystems*), conforme detalhado a seguir (Protocolo 1). Os reagentes utilizados nas reações foram fabricados pela *Leica Biosystems*.

1. Desparafinização com *Bond Dewax Solution* a 72 °C por 5 minutos;
2. Lavagem em álcool absoluto por 1 minuto, seguida de 4 minutos em água destilada;
3. Pré-tratamento do tecido (desmascaramento do RNA-alvo) com *Bond Enzyme 1* a 37 °C por 15 minutos;
4. Três lavagens em *Bond Wash Solution* por 2, 2 e 1 minuto, respectivamente;
5. Hibridização do RNA por 2 horas a 37 °C utilizando a sonda *BOND Ready-to-Use ISH HCMV Probe* PB0614;
6. Três lavagens em *Bond Wash Solution* por 2, 2 e 1 minuto, respectivamente;
7. Incubação com inibidor de peroxidase por 6 minutos à temperatura ambiente;
8. Três lavagens em *Bond Wash Solution* por 2, 2 e 1 minuto, respectivamente;
9. Incubação com anticorpo monoclonal de rato anti-fluoresceína AR0833 (*Leica Biosystems*) por 15 minutos à temperatura ambiente;
10. Três lavagens em *Bond Wash Solution* por 2, 2 e 1 minuto, respectivamente;
11. Incubação com reagente pós-primário por 8 minutos à temperatura ambiente, seguida de lavagem de 6 minutos em *Bond Wash Solution*;
12. Incubação com polímero de ligação por 8 minutos à temperatura ambiente, seguida de lavagem de 4 minutos em *Bond Wash Solution* e 1 minuto em água destilada;
13. Revelação da reação com DAB (3,3’-diaminobenzidina tetrahidroclorídrica) por 7 minutos à temperatura ambiente, seguida de lavagem de 2 minutos em água destilada;
14. Contracoloração com hematoxilina por 5 minutos à temperatura ambiente, seguida de lavagem de 1 minuto em *Bond Wash Solution* e 30 segundos em água destilada.

Para biópsias de enxerto renal (Tabela 2), outro protocolo foi empregado para reduzir danos decorrentes do tratamento enzimático e a intensidade de sinais de fundo. Todos os passos foram realizados usando o sistema automatizado BOND-MAX (*Leica Biosystems*), conforme as instruções do fabricante. As seguintes modificações foram implementadas (Protocolo 2):

- Após enxágue em água destilada, o pré-tratamento do tecido (recuperação do RNA-alvo) foi realizado utilizando *BOND Epitope Retrieval Solution* pH 9 por 30 minutos a 99 °C;
- Incubação das lâminas com inibidor de peroxidase por 30 minutos;
- Incubação das lâminas com inibidor de bloqueio proteico por 30 minutos à temperatura ambiente;
- Incubação com anticorpo monoclonal de rato anti-fluoresceína AR0833 (*Leica Biosystems*) por 11 minutos à temperatura ambiente;
- Desenvolvimento com DAB (3,3’-diaminobenzidina tetrahidroclorídrica) por 1 minuto à temperatura ambiente.

O procedimento automatizado de CISH levou aproximadamente 4 horas e 30 minutos. Após a conclusão do processo, as lâminas foram retiradas do equipamento, hidratadas em uma série graduada de etanol (4 vezes) e clareadas em xileno (3 vezes) antes da montagem em resina acrílica (Allklan, Allkimia) com lamínula.

As lâminas de tecido controle positivo e negativo de RNA seguiram o mesmo protocolo das amostras teste, com diferenças no passo 3, utilizando sondas de oligonucleotídeo conjugadas à fluoresceína específicas para os transcritos de mRNA positivos e negativos.

Essas lâminas foram analisadas separadamente em microscópio óptico por duas médicas patologistas (JMCOC e LSR) e uma bióloga (JVR). A positividade foi definida como imunorreatividade citoplasmática ou nuclear, granular, e de coloração marrom intensa. As células positivas foram quantificadas examinando-se toda a lâmina. A reprodutibilidade da coloração positiva foi avaliada por análise comparativa das três lâminas de cada caso do primeiro grupo (Tabela 1). Amostras que não exibiram as características especificadas foram consideradas negativas.

Ademais, as lâminas foram avaliadas de forma semiquantitativa, utilizando-se o seguinte sistema de pontuação: (0) quando não havia células positivas; (1) quando havia de 1 a 5 células positivas; (2) quando havia de 6 a 11 células positivas; e (3) quando havia mais de 12 células positivas. Toda a amostra foi examinada para determinar a pontuação final.

## Resultados

A preservação do RNA foi confirmada pelas reações positivas observadas com a utilização da sonda PB0785 *RNA Positive Control Probe* (Figura 1A). A especificidade da reação foi verificada pela ausência de resultados positivos com a utilização da sonda PB0809 *RNA Negative Control Probe* (Figura 1B). Todas as lâminas com tecido controle positivo apresentaram positividade (Figura 1C), enquanto nenhuma das lâminas de controle negativo apresentou positividade (Figura 1D). Reações positivas para HCMV foram detectadas com marcação granular, citoplasmática e nuclear nas células infectadas (Figura 2).

**Figura 1:** Verificação da integridade do RNA e controles padrão para a técnica de CISH para HCMV. A – Tecido palatino demonstrando expressão de RNA em todo o tecido, utilizando a sonda PB0785 *RNA Positive Control Probe*. B – Tecido palatino sem expressão de RNA em todo o tecido, utilizando a sonda PB0809 *RNA Negative Control Probe*. C – Tecido palatino com células apresentando expressão granular citoplasmática e nuclear de RNA citomegálico (Controle Positivo – Padrão). D – Tecido renal sem expressão de RNA citomegálico nas células (Controle Negativo – Padrão). Barra de escala: 20 µm.

**Figura 2:** Padrões de coloração por CISH de células positivas para HCMV em tecido palatino. A – Positividade citoplasmática (seta). B – Positividade nuclear (seta) e positividades granulares (cabeça de seta). Barra de escala: 20 µm.

Os resultados do primeiro grupo, composto por 10 amostras, estão apresentados na Tabela 3. A reação de CISH no grupo em questão mostrou-se reprodutível, com a mesma região apresentando positividade em todas as três lâminas testadas em dias diferentes (Figura 3).

**Tabela 3:** Comparação da análise quantitativa da hcmv utilizando as técnicas de ihq e cish

Caso	IHQ – Pontuação	CISH – Pontuação		
		teste 1	teste 2	teste 3
1	3	3	3	3
2	1	1	1	1
3	0	2	2	2
4	1	1	1	1
5	1	1	1	1
6	3	3	3	3
7	1	1	1	1
8	1	1	1	1
9	1	1	1	1
10	1	1	1	1
11	0	0	0	0

Nota – Pontuação: (0) nenhuma célula positiva; (1) de 1 a 5 células positivas; (2) 6 a 11 células positivas; e (3) mais de 12 células positivas.

**Figura 3:** Reprodutibilidade da CISH automatizada para detecção de HCMV. Reações triplicadas de hibridização *in situ* cromogênica (CISH) demonstrando detecção consistente da expressão de RNA do citomegalovírus (HCMV). A, B e C – Mesma região de uma biópsia de palato; D, E e F – Mesma região de uma biópsia esofágica, célula positiva (seta). Barra de escala: 50 µm.

Já os resultados das biópsias renais estão apresentados na Tabela 4. A reação por IHQ foi positiva em apenas uma amostra. A reação de CISH foi positiva em sete amostras e negativa em 13 das 20 amostras selecionadas. A comparação entre IHQ e CISH mostrou que células negativas na IHQ apresentaram positividade na CISH (Figuras 4 e 5). As modificações no protocolo de CISH (Protocolo 2) reduziram o sinal de fundo e melhoraram a qualidade geral da marcação (Figuras 4 B-C e E-F).

**Tabela 4:** Comparação da análise quantitativa do hcmv usando técnicas ihq e cish em biópsias renais

Caso	IHQ – pontuação	CISH - pontuação
12	0	0
13	0	0
14	0	0
15	0	0
16	0	0
17	0	0
18	0	2
19	0	3
20	0	3
21	0	0
22	0	0
23	0	0
24	0	3
25	0	0
26	0	2
27	0	3
28	0	0
29	0	0
30	0	0
31	1	3

Nota – Pontuação: (0) nenhuma célula positiva; (1) de 1 a 5 células positivas; (2) 6 a 11 células positivas; e (3) mais de 12 células positivas.

**Figura 4:** Análise comparativa de protocolos de CISH para detecção de HCMV em biópsias de palato e rim. A e D – Técnica IHQ positiva para expressão de proteínas de HCMV em células do palato (A) e negativa em biópsia renal D; B e E – Técnica CISH (Protocolo 1) positiva para expressão de RNA de HCMV no palato (B) e na biópsia renal – interstício do epitélio tubular renal (E); C e F – Técnica CISH (Protocolo 2) positiva para expressão de RNA de HCMV no palato (C) e na biópsia renal – interstício do epitélio tubular renal (F). Nota-se que, com o protocolo 2, o sinal de fundo da CISH é reduzido. Barra de escala: 20 µm.

**Figura 5:** Comparação das técnicas IHQ e CISH para HCMV em biópsias de enxerto renal. A e C – Técnica de IHQ negativa para expressão de proteínas de HCMV nas células renais. B e D – Mesmas regiões mostrando células positivas para RNA de HCMV pela técnica CISH. Barra de escala: 50 µm; detalhe: 20 µm.

Além disso, algumas células negativas para a marcação de HCMV por IHQ apresentaram positividade quando a CISH foi utilizada (Figura 5).

## Discussão

Este estudo teve como objetivo validar e implementar um protocolo automatizado de hibridização *in situ* (CISH) para a detecção de HCMV, utilizando amostras de diversos tipos de tecido, incluindo tecido renal de pacientes transplantados. A técnica selecionada foi a CISH com detecção de RNA durante a fase inicial da replicação viral. Essa escolha baseou-se na disponibilidade do equipamento necessário para a realização desse protocolo específico, bem como no fato de se tratar da única sonda comercialmente disponível no mercado brasileiro, à época do estudo, para a detecção de HCMV em tecidos incluídos em parafina, além de seu potencial como método complementar à técnica automatizada de IHQ, realizada no mesmo equipamento. Com base nos resultados obtidos, torna-se viável implementar a detecção automatizada de HCMV como parte do fluxo de trabalho diagnóstico de rotina. Embora a IHQ seja frequentemente considerada a técnica padrão para detectar HCMV em biópsias, a CISH oferece diversas vantagens, conforme demonstrado na Tabela 5.

**Table 5:** Comparação entre ihq e cish para detecção de hcmv em biópsias

	IHQ	CISH
Sensibilidade	++	++++
Custo	$$	$$$
Tempo (manual)	16h	16h
Tempo (automatizado)	5h	5h
Indicação	Suspeita de infeção	Suspeita de infeção IHQ negativa ou pontuação 1

A CISH, assim como a hibridização *in situ* (ISH), permite a avaliação de alterações genéticas, como variações no número de cromossomos, translocações ou ampliações, por meio da hibridização de sondas de DNA (ou RNA) marcadas com sequências complementares nos núcleos em interfase do tecido-alvo, sendo aplicável a tecidos fixados em formalina e incluídos em parafina^19^. A CISH também é comparável à hibridização *in situ* por fluorescência (FISH) no que diz respeito aos pré-tratamentos e protocolos de hibridização, diferindo no método de detecção; a CISH utiliza uma reação cromogênica baseada em peroxidase, semelhante à IHQ, em vez de marcadores fluorescentes. Sondas de RNA ou DNA conjugadas à fluoresceína são detectadas indiretamente usando um anticorpo conjugado à enzima (peroxidase). A reação enzimática com o substrato cromogênico (DAB) resulta em sinais permanentes marrons, visíveis à microscopia óptica padrão, permitindo o armazenamento prolongado das lâminas. Em contraste, a FISH requer interpretação sensível ao tempo, microscópio de fluorescência para leitura e apresenta menor durabilidade das lâminas, o que torna a CISH uma alternativa econômica^20^. A análise comparativa entre os dois métodos, FISH e CISH, foi bem apresentada e correlacionada por Hsi et al.^21^. Embora ambas as técnicas apresentem vantagens distintas, a CISH tem se mostrado cada vez mais prática, confiável e custo-efetiva em relação à FISH. Além disso, mostra-se adequada para o diagnóstico e a detecção de moléculas de RNA em diversos tipos de tecidos, incluindo HCMV, frequentemente associado a tecidos neoplásicos e lesões inflamatórias^10^. A CISH permite, ainda, a avaliação simultânea da amplificação gênica e da morfologia tecidual, com lâminas que podem ser armazenadas por longos períodos e visualizadas em microscopia óptica^19^.

A CISH automatizada é amplamente utilizada em Departamentos de Patologia^22,23,24^ para superar limitações do método manual, como o longo tempo de execução, a incubação durante a noite e a necessidade de envolvimento contínuo do técnico^17,22,23,25^. A automação na IHQ, ISH e CISH garante qualidade consistente, operações padronizadas, eficiência de custos, facilidade de uso e maior biossegurança^18^.

A sonda de oligonucleotídeo de HCMV conjugada à fluoresceína foi utilizada para detectar o HCMV por meio do RNA expresso durante a fase inicial da replicação viral. Nakajima et al.^26^ observaram que sondas de oligonucleotídeo de RNA são as mais sensíveis e adequadas para detectar baixos níveis de transcritos, o que explica a variação na marcação celular observada em nossos resultados, conforme mostrado na Tabela 2. Algumas amostras testadas apresentaram contagens celulares diferentes na CISH em comparação com a IHQ prévia, consistentes em todas as três reações. Outras amostras não apresentaram diferenças significativas na contagem de células entre as duas técnicas, possivelmente devido ao baixo número de cópias virais^7,16^.

O segundo protocolo, utilizando *BOND Epitope Retrieval Solution* pH 9 para o pré-tratamento do tecido (desmascaramento do RNA-alvo), foi validado (Figura 5) como alternativa para amostras potencialmente sensíveis à ação enzimática, devido a questões pré-analíticas, como a degradação tecidual observada na coloração por HE ou desafios na fixação e no processamento histológico^27,28^.

Dada a dimensão limitada das amostras de biópsia de enxerto renal e a necessidade de preservar a morfologia tecidual, um protocolo alternativo (Protocolo 2) foi avaliado. As modificações incluíram tempo prolongado de incubação para bloqueio da peroxidase endógena, adição de uma etapa de bloqueio proteico e redução do tempo de incubação com DAB. Esses ajustes foram necessários porque o tecido renal contém altos níveis de biotina endógena, que podem causar coloração de fundo e resultados falso-positivos após a aplicação do DAB^29,30^.

A marcação citoplasmática não característica (considerada negativa) em algumas amostras, juntamente com células positivamente marcadas, pode indicar rompimento de proteínas do capsídeo mal fixadas ou degradadas, com possível hibridização de RNA viral residual, conforme observado por Wolber e Lloyd^31^. No entanto, essas observações não interferiram no resultado geral da reação.

A CISH automatizada demonstrou boa reprodutibilidade, alta sensibilidade, com reações positivas em todas as amostras positivas por IHQ e ausência de marcação nos controles negativos, validando a técnica para implementação. Estudos de Todorovic´-Rakovic´^19^, Atabati et al.^32^ e Khaleghian et al.^33^ também confirmam que o método CISH é superior quando comparado à FISH e à IHQ, demonstrando maior eficácia, especificidade e sensibilidade.

É importante destacar que a infecção por HCMV é uma das principais causas de morbidade e mortalidade em pacientes imunocomprometidos e/ou imunossuprimidos, incluindo receptores de transplantes de órgãos sólidos, especialmente nos primeiros seis meses após o transplante devido à imunossupressão. O HCMV é a complicação mais comum em transplantes, com um risco cinco vezes aumentado para mortalidade global e onze vezes para óbito associado à infecção por HCMV. Portanto, a prevenção e o tratamento da infecção por HCMV são cruciais para o sucesso do transplante^34,35^ e estão associados ao aumento do risco de rejeição do enxerto^36,37^. No entanto, para confirmar a relevância clínica e o diagnóstico de nefrite viral por CMV, será crucial realizar um estudo ampliado com uma população maior de receptores de transplante renal, incluindo uma descrição detalhada da histologia renal e dos desfechos clínicos dos pacientes, juntamente com a detecção por CISH^38^.

O diagnóstico histopatológico preciso, utilizando técnicas mais sensíveis, como a CISH, pode indicar rejeição do órgão pós-transplante devido à infecção por HCMV ou sua associação. A padronização desse método possibilita sua integração às práticas laboratoriais de rotina e aumenta a confiabilidade diagnóstica, especialmente em casos de biópsias renais recorrentes, alinhando-se aos achados de Rimsza et al.^8^ e aos padrões internacionais^39^.

## Conclusão

Em nossos testes, a CISH superou a IHQ na detecção de células positivas para HCMV, especialmente em amostras com poucas células infectadas ou com padrões morfológicos atípicos. A CISH apresentou maior sensibilidade na identificação de células infectadas em estágio inicial, expressando RNA viral citoplasmático, independentemente da apresentação completa do vírus na célula, caracterizada por citomegalia e inclusões intranucleares. Embora variáveis como fixação, processamento histológico e tipo de tecido possam influenciar os resultados da hibridização *in situ*, o protocolo pode ser otimizado e adaptado para superar essas limitações técnicas potenciais.

A validação e a padronização da técnica automatizada de CISH, bem como sua implementação em contextos diagnósticos e de pesquisa para HCMV em biópsias renais e outros tipos de tecido, beneficiarão pacientes renais pós-transplante e outros pacientes com suspeita clínica e/ou morfológica de HCMV, aumentando a precisão do diagnóstico histopatológico. É importante destacar que este estudo teve como objetivo a validação do protocolo e a padronização da técnica automatizada de CISH. Estudos maiores, envolvendo o acompanhamento de pacientes transplantados, a detecção precoce potencial da infecção por HCMV e o prognóstico dos pacientes, ainda são necessários.

## Data Availability

Os conjuntos de dados gerados e/ou analisados durante o presente estudo estão disponíveis mediante solicitação razoável ao autor correspondente.
